# The key role of sphingolipid metabolism in cancer: New therapeutic targets, diagnostic and prognostic values, and anti-tumor immunotherapy resistance

**DOI:** 10.3389/fonc.2022.941643

**Published:** 2022-07-27

**Authors:** Run-Ze Li, Xuan-Run Wang, Jian Wang, Chun Xie, Xing-Xia Wang, Hu-Dan Pan, Wei-Yu Meng, Tu-Liang Liang, Jia-Xin Li, Pei-Yu Yan, Qi-Biao Wu, Liang Liu, Xiao-Jun Yao, Elaine Lai-Han Leung

**Affiliations:** ^1^ State Key Laboratory of Dampness Syndrome of Chinese Medicine, The Second Affiliated Hospital of Guangzhou University of Chinese Medicine (Guangdong Provincial Hospital of Chinese Medicine), Guangzhou, China; ^2^ Guangdong-Hong Kong-Macau Joint Lab on Chinese Medicine and Immune Disease Research, Macao, Macao SAR, China; ^3^ Dr. Neher’s Biophysics Laboratory for Innovative Drug Discovery/State Key Laboratory of Quality Research in Chinese Medicine/Macau Institute for Applied Research in Medicine and Health, Macau University of Science and Technology, Macao, Macao SAR, China; ^4^ Department of Oncology, Luzhou People’s Hospital, Luzhou, Sichuan, China; ^5^ Cancer Center, Faculty of Health Science, University of Macau, Macao, Macao SAR, China; ^6^ MOE Frontiers Science Center for Precision Oncology, University of Macau, Macao, Macao SAR, China; ^7^ Breast Surgery, Zhuhai Hospital of Traditional Chinese and Western Medicine, Zhuhai, China

**Keywords:** sphingolipid metabolism, enzymes, cancer, immunotherapy, anticancer

## Abstract

Biologically active sphingolipids are closely related to the growth, differentiation, aging, and apoptosis of cancer cells. Some sphingolipids, such as ceramides, are favorable metabolites in the sphingolipid metabolic pathway, usually mediating antiproliferative responses, through inhibiting cancer cell growth and migration, as well as inducing autophagy and apoptosis. However, other sphingolipids, such as S1P, play the opposite role, which induces cancer cell transformation, migration and growth and promotes drug resistance. There are also other sphingolipids, as well as enzymes, played potentially critical roles in cancer physiology and therapeutics. This review aimed to explore the important roles of sphingolipid metabolism in cancer. In this article, we summarized the role and value of sphingolipid metabolism in cancer, including the distribution of sphingolipids, the functions, and their relevance to cancer diagnosis and prognosis. We also summarized the known and potential antitumor targets present in sphingolipid metabolism, analyzed the correlation between sphingolipid metabolism and tumor immunity, and summarize the antitumor effects of natural compounds based on sphingolipids. Through the analysis and summary of sphingolipid antitumor therapeutic targets and immune correlation, we aim to provide ideas for the development of new antitumor drugs, exploration of new therapeutic means for tumors, and study of immunotherapy resistance mechanisms.

## Introduction

**Figure 1:**
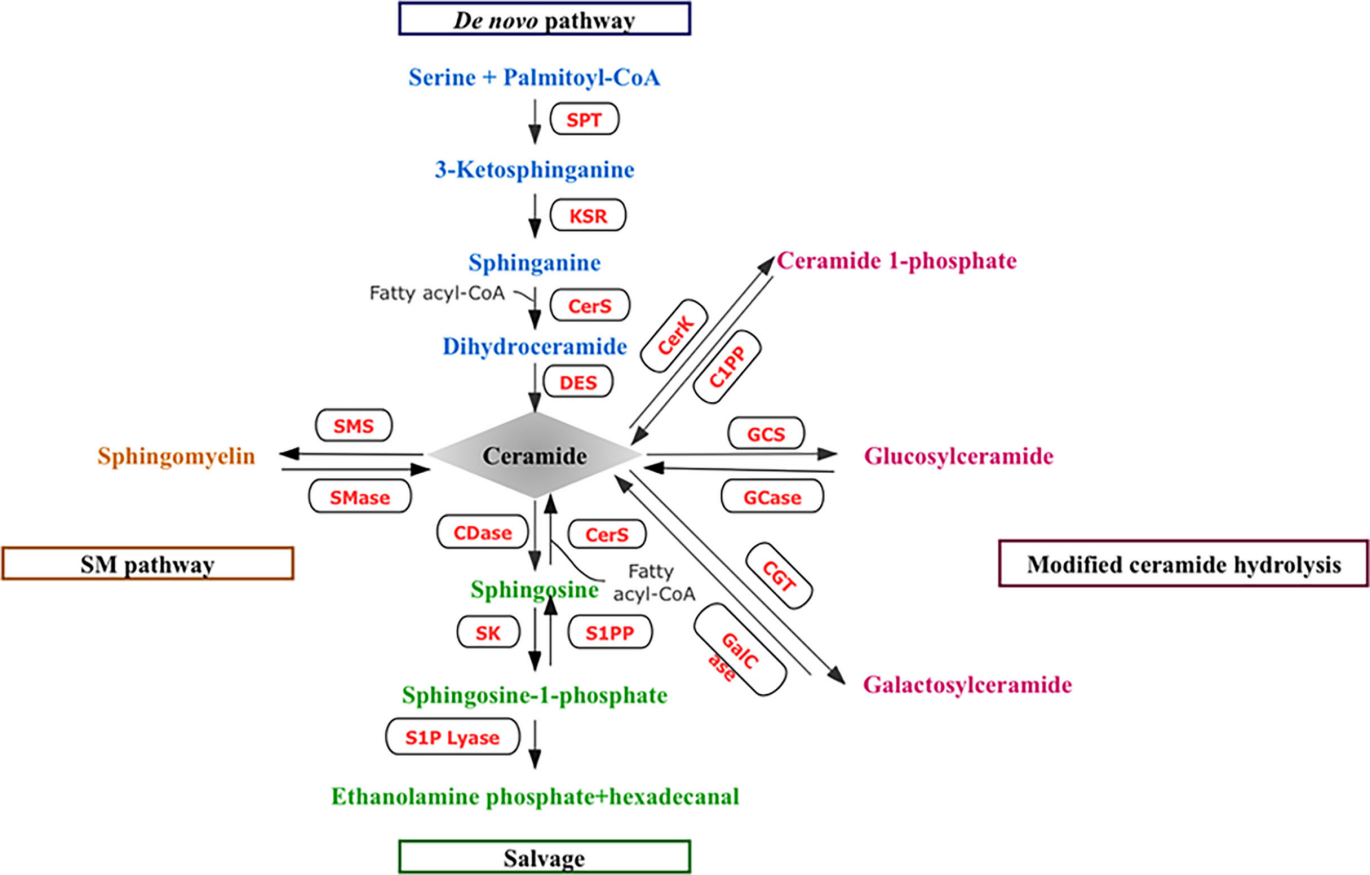
Metabolic pathway of sphingolipids.

Over the last 2–3 decades, we have gained a great understanding of the structural biodiversity, cell biology, metabolisms, and pathology of sphingolipids. Various molecules from this lipid family participate in a variety of cellular functions in health and disease (1). As shown in **Figure 1**, ceramides constitute the hub of sphingolipid metabolism and participate in the synthesis and catabolic metabolism of sphingolipids. Ceramides can be generated through several different mechanisms. Endogenous ceramides can be synthesized *via de novo* pathway following the action of a range of enzymes, such as serine palmitoyltransferase (SPT), ceramide synthase (CerS), and dihydroceramide desaturase (DES). Ceramides may also be produced through the decomposition of membrane sphingomyelins (SMs) or the degradation of complex sphingolipids under the action of sphingomyelinases (SMase) and glucosylceramidases, respectively. In addition, sphingosine, a product of sphingolipid catabolism, can be reacylated by CerS, resulting in the production of ceramides. Once produced, ceramides can briefly accumulate or be converted into different, sphingolipids, such as ceramide 1-phosphate (C1P), sphingosine 1-phosphate (S1P), and glucosylceramide (GlcCer). Ceramide can also be reversed into SMs, that is, phosphorylcholine and ceramide generate SMs under the action of sphingomyelin synthetase (SMS). In addition, ceramides can be glycosylated by different enzymes of the Golgi Apparatus into glycosphingolipids (GSLs). Moreover, sphingolipids are also glycosylated in the lysosomes (2). The phosphorylation of ceramide also occurs in Golgi Apparatus by CerK (3) (**Figure 1**).

The mortality rate caused by malignant tumors has nearly doubled in the last two decades (4), and cancer remains the highest cause of mortality worldwide. Sphingolipids and tumors are closely related. The correlation between them has received increasing attention from researchers. Sphingolipids, among all the reasons for the pathogenesis of cancer researched thus far, play a critical role in the initial and developmental stages of cancer. Sphingolipids serve as bioeffectors that coordinate all aspects of cancer biology, including apoptosis, cell proliferation, and cell migration. Ceramide and S1P are important sphingolipids but have opposite effects. Ceramide promotes cancer cells apoptosis, blocks cell growth; S1P promotes cancer cells survival, enhancing cell proliferation, angiogenesis. The dynamic balance of these two opposing sphingolipids is called the “Sphingolipid rheostat” (5). The sphingolipid-regulated processes are critical for the development, progression, metastasis, and drug resistance of different types of cancers (1).

## The value of sphingolipid metabolism in cancer

### The expression level of sphingolipids related to cancer diagnosis and prognosis

Sphingolipids are also expressed differently in various tumor subtypes. Recent studies have shown that some classes of ceramides may be elevated in certain human malignancies. For example, C16:0 and C24:1 ceramides are up-regulated in node-positive pancreatic tissues (6). Similar observations on up-regulated sphingolipids have been found for B16 melanoma, murine sarcoma 180, and Lewis lung carcinoma (7); human head and neck squamous cell carcinoma (HNSCC) (8); rat nephroma-RA (9); and breast cancer (10). By contrast, colon cancer (11), ovarian carcinoma (12), and human astrocytoma (13) have lower ceramide levels than their corresponding healthy organizations. in certain human malignancies, since in some works depending on the species, their levels are decreased. Another published report found that C18-Cer is selectively down-regulated in HNSCC relative to in nonsquamous tumor tissues (14).

Then, in the downstream of sphingolipid pathways, the activity of SPHK1/S1P plays an important role in many cancers, and SPHK1 has been proven to be highly expressed in a variety of cancers (15–17). An investigation has been applied to test the tissue samples from prostate cancer patients and healthy individuals. The results proved that SPHK1 expression is 2-fold higher in tissues from patients than in healthy individuals (18). This phenomenon had also been observed in breast cancer. For example, a previous work performed microarray analysis on various subtypes in 1269 breast cancer samples and found increased SPHK1 expression in the tissues of patients with cancer (19). Another study showed that, in breast cancer, SPHK1 and ceramide galactosyltransferase (UGT8) are highly expressed in ER-negative tumors, and dihydroceramide synthases (LASS4 and LASS 6), acid ceramidase (ACDase), and glucosylceramide synthase (GCS) are highly expressed in ER-positive samples (19). Moreover, numerous other cancers have high SPHK1 expression (20–23). These cancers include oral squamous cell carcinoma (24), gastric cancer (15), hepatocellular carcinoma (HCC) (16, 17), glioblastoma multiforme (20), prostate cancer (21), esophageal cancer (22), and HNSCC (23) (**Table 1**). All the above pieces of information show that the cell-transformation-related alterations in sphingolipid metabolism are dependent on cancer types. We have summarized the abnormal sphingolipid metabolism of different cancers in **Table 1**. Changes in sphingolipid metabolism contribute to the progress of cancers and provide useful targets for the development of new targeted therapies. Moreover, some sphingolipids could be used as biomarkers in cancer diagnosis. The latest data have shown that in patients with breast cancer, the increase in C16-Cer is associated with metastatic lymph node (LN) status (10), suggesting that C16-Cer has metastatic potential in the clinic. Interestingly, an investigation on colorectal cancer (CRC) tissue has suggested that CRC shows increased amounts of S1P, Sph, and C14-Cer and markedly reduced amounts of C18-Cer and C20-Cer. The circulating contents of C16:0-Cer, C18:1-Cer, C20-Cer, and C24:1-Cer in more advanced CRC were higher than those in early lesions (32). These results suggest that early lesions can be distinguished from advanced CRC through the combined determination of the plasma concentrations of several ceramides. In addition to being the diagnostic biomarkers, sphingolipids could also be used as biomarkers in cancer prognosis. Clinical studies have reported that the expression levels of SPHK and S1P are associated with patient survival and cancer metastasis (22, 23). An online database of gene expression and clinical data from 1928 patients with NSCLC has showed that although the high expression of SPHK1 mRNA is significantly correlated with worsened overall survival (OS), the high expression of SPHK2 mRNA is correlated with improved OS (23). Interestingly, in esophageal cancer, the high expression of SPHK1 is associated with poor 5-year OS and increased SPHK1 levels are significantly correlated with LN metastasis; therefore, SPHK1 is a potential biomarker for outcome prediction in clinical practice (22). A meta-analysis has shown that increased SPHK1 expression is associated with poor prognosis in human cancers and could be a promising prognostic marker and therapeutic target in patients with malignancies (33). Despite SPHK1 and SPHK2 are highly homologous, different subsets of SPHK execute different physiological roles in cancers. However, both subtypes have important roles in inducing cancer cell survival (34, 35).

**Table 1:** Summary of sphingolipid biomarkers in various cancers.

Type of cancer	Sphingolipid/Enzyme	Sample	Result	Refs
Breast cancer	SMS2	Basal-like breast cancer and luminal-like breast cancer (n=522)	Patients with high SMS2 expression have poorer prognosis	(25)
	C16:0-Cer, C24:1-Cer, C24:0-Cer	Malignant breast tumor tissues (n=43) normal tissues (n=21)	•The levels of C16:0-Cer, C24:1-Cer and C24:0-Cer were significantly raised in malignant tumors as compared with benign and normal tissue •C16:0-Cer raised level was associated with a metastatic LN status	(10)
	CerK	Breast tumor tissues (n≥2200)	•The elevated CerK expression was associated with an increased risk of recurrence in women with breast cancer	(26)
	SPHK1,UGT8, GCS,LASS4,LASS6,ACDase	Different subtypes of breast cancer tumor samples (n=1269)	•Patients with high SPHK1 expression have poorer prognosis •SPHK1 and UGT8 displayed higher expression among ER negative tumors •GCS, LASS4, LASS 6 and ACDase were higher expressed in ER positive samples	(19)
	S1P	Breast cancer tissue samples (n=35)	•Levels of S1P in breast cancer tissues were significantly higher in patients with high white blood cell count in the blood than those patients without •S1P levels were lower in patients with human epidermal growth factor receptor 2 over-expression and/or amplification than those patients without •Cancer tissues with high pSPHK1 expression showed significantly higher levels of S1P than cancer tissues without •Patients with lymph node metastasis showed significantly higher levels of S1P in tumor tissues than the patients with negative nodes	(27)
	Sph, DHCer, S1P	Breast cancer tissue samples (n=7)	Sphingosine, dihydrosphingosine, and S1P levels were significantly higher in human breast tumor tissue IF than in the normal breast tissue IF	(28)
	SPHK1	Breast cancer tissue samples (n=65)	•Levels of SPHK1 in TNBC patients were significantly higher than levels in other patients with other breast tumors •The expression of SPHK1 was positively correlated with poor OS and PFS, as well as poor response to 5-FU and doxorubicin	(29)
		Breast cancer tissue specimens (n=120)	The level of SPHK1expression in the breast cancer tissue was significantly higher in patients with estrogen and progesterone negative receptors, compared to the ones without them	(27)
	GCS	Affymetrix microarray experiments from primary breast cancer patients (n=1681)	•Expression of GCS was associated with a positive ER status •High GCS expression was also associated with poor outcomes in ER+ tumors	(30)
OSCC	SPHK1	OSCC tissue samples (n=69)	Patients with OSCCs with high SPHK1 expression showed higher invasive grades and unfavorable survival rates	(24)
Gastric cancer	SPHK1	Gastric cancer tissue samples (n=175)	•Levels of SPHK1 mRNA and protein were higher in gastric cancer cell lines than in normal gastric epithelial cells •Patients with higher SPHK1expression had shorter overall survival time, whereas those with lower SPHK1 expression survived longer	(15)
HCC	SPHK1	HCC tissue samples (n=21)	•The SPHK1 expression levels were identified to be significantly upregulated in HCC tissue compared with that in adjacent normal tissue samples •High SPHK1 expression correlated with shorter overall survival times in patients with HCC	(16)
		HCC tissue samples (n=127)	•The expression of SPHK1 in HCC tissue was revealed to be significantly higher than in normal tissue •SPHK1 expression was significantly associated with tumor size, tumor stage and histological differentiation •The patients with low SPHK1 expression had higher OS and recurrence-free survival rates compared with patients with high SPHK1 expression	(17)
Prostate cancer	SPHK1	Prostate cancer tissue samples (n=30)	•A signifificant 2-fold increase in SPHK1 enzymatic activity was observed in cancer •The upper quartile of SPHK1 activity was associated with higher PSA, higher tumor volumes, higher rates of positive margins and surgical failure than the lower three quartiles	(20)
	S1P,SPHK1	Patients with localised, locally advanced, or metastatic PCa (n=88) Patients with age-matched controls with BPH (n =110) Patients with young healthy males with the very small chance of having PCa foci (n = 20)	•Levels of circulating S1P were significantly higher in healthy subjects and patients with BPH than in patients with PCa •Died patients’ circulating S1P levels were significantly lower than in the surviving patients •Circulating S1P levels were an early marker of PCa progression to hormonal unresponsiveness and correlated with PSA levels and lymph node metastasis •The decrease in circulating S1P during PCa progression may stem from a highly significant downregulation of erythrocyte SPHK1 activity	(21)
Esophageal cancer	SPHK1	Thoracic squamous cell esophageal cancer specimens (n=177)	•Among 177 esophageal cancer patients, 127 (72%) were defined as being SPHK1-positive •SPHK1 expression status was a significant factor contributing to lymph node metastasis and poorer 5-year overall survival	(22)
Colon caner	S1P	sporadic CRC patients (n=10) CAC patients (n=10)	pSPHK1 expression to be more prevalent in CAC patients and to have a higher immunohistochemistry score than in sporadic CRC patients	(31)
	Cer,Sph,S1P,SPA,SPT,CerS1,CerS5	Consecutive adult patients with primary CRC (n=45)	•The highest content in CRC tissue was found for C16:0-Cer (80.36% of total ceramide) •The highest content (and higher than in normal tissue) was demonstrated for C24:0-Cer and Sph •CRC tissue showed increased amounts of S1P, SPA, and C14:0-Cer •CRC tissue showed significantly lower C18:0-Cer and C20:0-Cer contents in the tumor •SPT, CerS1, and CerS5 content in CRC tissue was lower than in normal intestinal tissue •A higher circulating content of C16:0-Cer, C18:1-Cer, C20:0-Cer and C24:1-Cer was demonstrated in more advanced colorectal cancer compared to early stages lesions	(32)
NSCLC	SPHK1,SPHK2,SGPL1	gene expression and clinical data of NSCLC patients (n=1928)	•High SPHK1 mRNA expression was significantly correlated to worse OS •High SPHK2 or SGPL1 mRNA expression was in favor of better OS	(23)

SMS2, sphingomyelin synthetase 2; C14:0-Cer,C14:0-ceramide;C16:0-Cer, C16:0-ceramide; C18:0-Cer,C18:0-ceramide;C18:1-Cer,C18:1-ceramide; C24:0-Cer, C24:0-ceramide; C24:1-Cer, C24:1-ceramide; SPHK1, sphingosine kinase 1; UGT8, ceramide galactosyltransferase; GCS, glucosylceramide synthase; LASS4/LASS6,dihydroceramidsynthases;ACDase, acid ceramidase;S1P, sphingosine 1-phosphateSph;DHCer, dihydroceramide; Cer, ceramide; Sph, sphingosine; SPA, sphinganine; SPT, serine-palmitoyltransferase; CerS1, ceramide synthase 1; CerS5, ceramide synthase 5; SPHK2, phingosine kinase 2; SGPL1, S1P lyase; ER, endoplasmic reticulum;IF, interstitial fluid; TNBC, triple negative breast cancer;LN, lymph node;OS, overall survival;PFS, progression-free survival;5-FU, 5-Fluorouracil;OSCC, oral squamous cell carcinoma;HCC, hepatocellular carcinoma; PSA, prostate specific antigen; CRC, colorectal cancer; CAC, colitis-associated cancer; NSCLC, non-small cell lung cancer.

### The function of sphingolipids in cancer

An increasing number of studies have explored the correlations between sphingolipids and tumors. The dynamic balance among sphingolipids maintains normal biological functions. In cancer, however, this balance can be disrupted. A growing body of evidence shows that in cancer, the levels of bioactive sphingolipids are altered. Thus, bioactive sphingolipids harbor the potential to act as important cancer biomarkers for the determination of disease progression (36). Given the different expression levels of metabolites in different cancers, the same sphingolipids could execute opposite roles in different cancers.

Ceramides are one of the most studied sphingolipids in cancers due to their role in cell differentiation and death. Diverse ceramides produce different lengths of ceramide chains that have unique and important biological functions *in vivo*. Ceramides can induce apoptosis through the mitochondrial pathway by changing the mitochondrial ultrastructure and reducing mitochondrial function and membrane potential, thus ultimately inducing apoptosis (37). For example, C18-Cer could modulate telomerase activity and mitochondrial dysfunction-induced apoptotic cell death in HNSCC (14), and C16-Cer could induce ER stress-mediated apoptosis in lung cancer (38). Ceramides can also cause cell death *via* autophagy. In liver and nasopharyngeal cancers, ceramides could induce autophagy through the regulation of Beclin-1 (39). Dihydroceramide could also activate the unfolded protein response that leads to cytotoxic autophagy in cancer cells (40). In colon cancer, ceramide inhibits cell proliferation and migration by down-regulating IL-10, STAT3, and NF-kB expression (41). Another published report has shown that in breast cancer, the overexpression of C16-Cer reduces the phosphorylation of Akt/mTOR and ERK (42). Ceramides have numerous mechanisms for cell death induction. Instead of inhibiting cell growth as mentioned above (42), C16-Cer protects against ER stress-induced apoptosis and enhances tumor development and growth in HNSCC (43). Similarly, ceramide has an opposite effect in colon cancer that is distinct from their effect in gallbladder cancer (44). C24-Cer induces cell proliferation and migration by binding to PIP4K2C to facilitate mTOR complex formation and activation (45) (**Table 2**).

**Table 2:** Sphingolipid metabolites and enzymes and their key cellular functions in cancer.

Cellular process	Cancer type		Cell lines	Mechanism of action	Refs
***Ceramide (Cer)***					
↑ Apoptosis	HNSCC		UM-SCC-22A cells	C18-ceramide inhibited cell growth by modulating telomerase activity and mitochondrial dysfunction-induced apoptotic cell death	(13)
	Lung cancer		A549, H157 and H1650 cells	CerS6/C16-ceramide activated ATF6 by releasing ca^2+^ from ER stores and induced ER stress-mediated apoptosis in squamous cell carcinomas	(38)
	Breast cancer		MCF-7 and MDA-MB-231 cells	C2-ceramide induced high cytotoxicity in MDA-MB-231cells by targeting mutant p53 expression C2-ceramide triggered senescence-signaling transduction in MCF-7 cells and resulted in MCF-7 cells escaping from C2-ceramide-induced apoptosis	(46)
↑Autophagy	Liver cancer、 Nasopharyngeal cancer		Hep3B and CNE2 cells	Ceramide induced Beclin-1- dependent autophagic cell death, which is mediated by JNK	(39)
	Breast cancer		MCF-7 cells	Increased long-chain ceramides through the downregulation of CerS2 has been shown to arrest growth with activation of PKR-like ER kinase	(47)
↑ Cell growth	HNSCC		UM-SCC-1 cells	CerS6/C16-ceramide protected against ER stress-induced apoptosis and enhanced tumor development and growth in HNSCC	(48)
↓ Cell growth	Breast cancer		MCF-7 cells	C16-ceramide generated by CerS6 overexpression reduced phosphorylation of Akt/mTOR and ERK	(42)
↑Cell proliferation ↑Cell migration	Gallbladder cancer		GBC-SD and NOZ cells	C24-Ceramide bound to PIP4K2C to facilitate mTOR complex formation and activation	(49)
↓ Cell proliferative ↓ Cell migratory	Colon cancer		CT-26 and MC-38 cells	Cer attenuated expression levels of IL-10 in colorectal cancer cells co-cultured with M2 macrophages and downregulated STAT3 and NF-kB expression	(41)
***Dihydroceramide (DHCer)***					
↑Autophagy	Pancreatic cancer、 Lung cancer		MiaPaca2 and A549 cells	Long chain dihydroceramides caused ER stress and activation of the UPR that ultimately lead to cytotoxic autophagy in cancer cells	(40)
***Sphingomyelin (SM)***					
↑Cell cycle arrest	Glioblastoma		U118 glioma cells	Increased SM inactivated the MAPK pathway. Through cross-talk, the inhibition of this pathway can impair the PI3K/Akt pathway and consequently the cell cycle	(50)
***Sphingosine (Sph)***					
↓Cell proliferation	Intestinal adenoma		RIE cells	Sphingosine downregulated Cdk4 expression and phosphorylation of phospho-Rb	(34)
***Ceramide 1-phosphate (C1P)***					
↑Inflammatory	Lung cancer		A549 cells	C1P has been shown to induce arachidonic acid release and is regulated by the direct binding between C1P and cPLA2	(35)
↑Cell proliferation	Lung cancer		A549 cells	Treatment of A549 cells with low concentrations of C1P (0.5–1 μM) increased cells proliferation	(51)
↑Apoptosis	Lung cancer		A549 cells	Treatment of A549 cells with concentrations of C1P of 5 μM markedly increased the number of cells apoptosis	(51)
↑Cell migratory	Pancreatic cancer		PANC-1 and MiaPaCa2 cells	C1P increased pancreatic cancer cell migration and invasion	(52)
***Sphingosine-1-phosphate (S1P)***					
↓ Apoptosis	Lymphatic cancer		Jurkat cells	S1P prevented apoptosis by inhibiting the translocation of cytochrome c and Smac/DIABLO from mitochondria to the cytosol induced by anti-Fas, TNF, serum deprivation, and short- chain ceramide	(53)
↑Cell migratory	Multiple myeloma		myeloma cells	S1P up-regulated myeloma cell adhesion mediated by α4β1 and transendothelial migration stimulated by CXCL12, suggesting that the cooperation of S1P and CXCL12 plays a role in MM cell progression	(54)
	Ovarian cancer		OVCAR3 cells	S1P stimulated chemotaxis and invasion of ovarian cancer cell in a receptor-dependent fashion that involved activation of ERK, AKT and p38	(43)
↑Cell metastasis	Liver cancer		HepG2 cells	S1P induced HCC metastasis via establishing an MMP-7/syndecan-1/TGF-β1 autocrine loop	(55)
	Breast cancer		MCF7 cells	S1P can rapidly up-regulate the expression of SNAI2 in breast cancer cellsviathe activation of cognate receptors S1P2 and S1P3	(56)
↓Melanin synthesis	Melanoma		Mel-Ab cells	S1P reduced melanin synthesis by ERK activation, MITF phosphorylation at Ser73 and degradation by the proteasome	(57)
↑Cell proliferation	Gastric cancer		MKN28 and MKN74 cells	S1P induced rapid and transient tyrosine phosphorylation of EGFR and c-Met	(58)
	Liver cancer		HepG2 and SMMC7721 cells	S1P augmented the proportion of cells in S phase of the cell cycle that might translate to enhance HCC cell proliferation and inhibit the cell apoptosis via syndecan-1	(59)
***Ceramide kinase (CerK)***					
↓Cell migratory ↓Cell metastasis		Lung cancer	A549 cells	CerK regulated the activity of Rac1 and overexpression of CerK inhibited lamellipodium formation	(60)
↑Cell migratory ↑Cell invasion		(Metastatic) Breast cancer	MDA-MB-231 and MCF-7 Cells	CerK activated PI3K and Akt signaling in metastatic cells	(61)
***Sphingomyelinase (SMase)***					
↑Apoptosis		Lymphatic cancer	HuT78 cells	ASMase-mediated pathway contributed to CD95-induced apoptotic signal	(62)
		Lung cancer	H1299 cells	NSMase generated ceramide was found to initiate apoptotic cell death upon overexpression of p53 and induce apoptosis	(63)
		Breast cancer	MCF-7 cells	The enforced mitochondrial targeting of NSMase in MCF7 cells resulted in mitochondrial ceramide increase that caused cytochrome c release and apoptotic cell death	(64)
***Sphingomyelin synthetase (SMS)***					
↑EMT ↑Cell migratory ↑Cell invasion		Breast cancer	MCF-7 and MDA-MB- 231 cells	SMS2 increased the expression of TGF- β1 by upregulating SM, which subsequently activated the TGF-β/Smad signalling pathway and promoted EMT in breast cancer cells, thus increasing the migration and invasiveness of breast cancer cells	(65)
↓Cell death		Lymphatic cancer	Jurkat cells	Overexpression of SMS1 protected cells from FasL-induced ceramide generation and cell death	(66)
***Sphingosine-1-phosphate (SPL)***					
↑Apoptosis		Colon cancer	HEK293 cells	SPL promoted apoptosis via p53 and p38-dependent pathway	(67)
***Sphingosine kinase 1 (SPHK1)***					
↑Autophagy		Colon cancer	HT-29 cells	Activation of the SPHK1/ERK/p-ERK pathway promotes autophagy in colon cancer	(68)
↑Cell invasion ↑Cell metastasis		Esophageal cancer	EC9706 cells	SPHK1 was involved in upregulation of EREG and AREG through enhancing EGFR phosphorylation to promote invasion	(69)
		Liver cancer	HepG2 cells	SPHK1 induced the EMT by accelerating CDH1/E-cadherin lysosomal degradation	(70)
		Prostate cancer	PCa cells	LPS induced the S225 phosphorylation of SPHK1 and the translocation of SPHK1 to plasma membrane, leading to the production of S1P, ERK1/2 and matriptase activation via S1P4	(71)
↑Cell proliferation		Intestinal adenoma	RIE cells	The overexpression of SPHK1 resulted in an enhancement in the G1/S transition of the cell cycle in RIE cells	(34)
		Breast cancer	MCF-7, SKBR3, MDA-MB-468 ,and HCC38 cells	The SPHK1-S1P axis is hyper-activated in breast CSCs and promoted cell survival in both breast CSCs and non-CSCs by suppressing STAT1 expression	(72)
↓Apoptosis		Erythroleukemic	HS1 cells	SPHK1 activated the ERK1/2 and PI3K/AKT pathways	(73)
↑cell migratory		Colon cancer	Caco2, HT29, RKO and HCT116 cells	SPHK1 increased the expression of Slug, vimentin, N-cadherin and FAK	(29)
			RKO and HT29 cells	SPHK1 promoted the migration and metastasis of colon cancer by inducing EMT mediated by the FAK/AKT/MMPs axis	(74)
↑Cell metastasis		Breast cancer	E0771 breast cancer cells	The upregulation of SPHK1, formation of S1P, and subsequent activation of S1P1 lead to persistent activation of survival signaling and STAT3 in a malicious feed-forward amplification loop critical	(75)
			Human breast cancer cells	SPHK1 promoted metastasis of TNBC through S1P/S1P3/Notch signaling pathway	(29)
↑Drug resistance		Colon cancer	SK-Hep1 and HCCLM3 cells	SPHK1 promoted oxaliplatin resistance of HCC cells via modulation of the Akt/GSK3β signaling pathway	(16)
***Sphingosine kinase 2 (SPHK2)***					
↑Cell survival		Leukaemia	Human NKL cells	SPHK2 inhibition downregulated pro-survival Mcl-1 protein through proteasomal degradation	(76)
***Glucosylceramide synthase (GCS)***					
↑Drug resistance		Breast cancer	NCI/ADR-RES cells	GSLs, in particular of globo series GSLs mediate gene expression of MDR1 through cSrc and β-catenin signaling	(77)

HNSCC, head and neck squamous cell carcinoma; CerS2, ceramide synthase 2; CerS6, ceramide synthase 6; ATF6, activating transcription factor 6; ER, endoplasmic reticulum; JNK, c-Jun N-terminal kinase, PKR, double-stranded RNA-dependent protein kinase; Akt, protein kinase B; mTOR, mammalian target of rapamycin; PIP4K2C, phosphatidylinositol-5-phosphate 4-kinase type 2 gamma; IL-10, Interleukin 10; STAT1, signal transducerand activator of transcription 1; STAT3, signal transducerand activator of transcription 3; NF-kB, nuclearfactor-kappaB; UPR, unfolded protein response; MAPK, mitogen-activated protein kinase; PI3K, the phosphatidylinositol3-kinase; Cdk4, cyclin dependent kinase 4; cPLA2, cytosolic phospholipase A2; Smac, second mitochondria-derived activator of caspase; DIABLO, direct IAP binding protein with low pI; TNF, tumor necrosis factor; MM, multiple myeloma; ERK, extracellular regulated protein kinases; HCC, hepatocellular carcinoma; MMP-7, matrix metallopeptidase 7; TGF-β1; transforming growth factor-β1; S1P2, sphingosine-1-phosphate receptor 2; S1P3, sphingosine-1-phosphate receptor 3; S1P4, sphingosine-1-phosphate receptor 4; MITF, microphthalmia transcription factor; c-Met, c-Mesenchymal-epithelial transition factor; SMS1, sphingomyelin synthetase 1; SMS2, sphingomyelin synthetase 2 ; EMT, epithelial-mesenchymal transition; EGFR, anti-epidermal growth factor receptor; CDH1, cadherin 1; LPS, Lipopolysaccharide; CSC, cancer stem cell; FAK, focal adhesion kinase; TNBC, triple negative breast cancer; Mcl-1, myeloid cell leukaemia-1; GSL, glycosphingolipid; MDR1, multidrug resistance 1. ↑ means the cellular activity or process increased in the corresponding study, and ↓means the activity or process decreased.

In contrast to ceramides, S1P can modulate multifunctional biological activities in cancer, such as proliferation, migration, inflammation, and angiogenesis. As proposed by Hanahan and Weinberg, S1P is involved in all features of cancer, including sustained proliferative signaling, the evasion of growth inhibitors, resistance to apoptosis, the achievement of uncontrolled replication, angiogenesis, the activation of invasion and metastasis, the reprogramming of energy metabolism, and the evasion of the immune response (78). The generation of S1P from sphingosine is catalyzed by two SPHK isoforms, namely, SPHK1 and SPHK2. S1P receptors are called S1PR. S1P binds to five high-affinity G-coupled receptors with different tissue distribution patterns: S1PR 1–5. The specific role of S1P is dominated by the expressed S1PR subtype (44).

Similar to vascular endothelial growth factor, S1P has been reported to increase vascular permeability. The mechanism of activation involves the activation of the S1PR2 subtype and requires Rho/ROCK/PTEN signal transduction (79). In lymphatic cancer, S1P could prevent apoptosis by inhibiting the translocation of cytochrome c and Smac/DIABLO from the mitochondria to the cytoplasm (53). S1P has also been shown to influence the metastasis and migration of cancer cells. For example, S1P could induce HCC metastasis *via* establishing the MMP-7/syndecan-1/TGF-β1 autocrine loop (55). Moreover, it could stimulate the chemotaxis and invasion of ovarian cancer cells in a receptor-dependent manner that involves the activation of ERK, AKT, and p38 (43).

### The function of sphingolipid enzymes in cancer

The abundance of sphingolipids is controlled by metabolic enzymes, which have a crucial role in regulating cancer cell death or survival as summarized in **Table 2**. Thus, we next focus on the enzymes participating in cancer physiology. Some enzymes have been proven to be capable of enhancing ceramide synthesis and break down S1P to promote cancer cell death. These enzymes include SMase, SPT, CerS, DES, and S1P lyase (SPL), which need therapeutic activators (1). On the other hand, some enzymes that could eliminate and metabolize ceramides, such as CerK, CDase, SPHK, and SMS, which need therapeutic inhibitors.

SMase is a sphingolipid enzyme that has been widely reported. It can be divided into three types in accordance with its optimal pH-dependent activity: acid SMase (ASMase), neutral SMase (NSMase), and alkaline SMase (alk-SMase) (45). Many pieces of evidence had suggested that the activation of ASMase is a necessary step for the initiation of apoptosis in stress-induced ceramide elevation (62). NSMase has also been reported to cause apoptosis in cancer (63, 64). In addition, the lack of alk-SMase has been shown to be capable of significantly increasing colon tumorigenesis (80).

Another one, SPHK1, can promote proliferation and migration by promoting JAK/STAT activation and up-regulating S1PR1 expression (81). SPHK1 promotes triple negative breast cancer (TNBC) translocation through the S1P/S1PR3/Notch signaling pathway (29). Convincing evidence indicates that the activation of SPHK1 contributes to cancer progression and tumor growth and impairs cancer cell apoptosis *via* suppressing STAT1 expression (72), as well as activating the ERK1/2 and PI3K/AKT pathways (29) Many studies have demonstrated that the activation of SPHK1 can induce cancer cell migration, and that the SPHK1/S1P axis enhances the metastatic potential of cancer cells. Mechanism studies have reported that SPHK1 increases the expression of Slug, vimentin, N-cadherin, and FAK in colon cancer (82) Notably, the active interaction of SPHK1 or S1P with ERK pathway promotes autophagy in colon cancer (68).

CerK is a calcium ion-dependent enzyme that catalyzes the production of C1P in cells. Intracellular C1P can perform a variety of biological functions, including the stimulation of tumor cell growth, the stimulation of VEGF release, the inhibition of tumor cell apoptosis, the promotion of tumor inflammation, the regulation of angiogenesis, and the stimulation of tumor cell migration (83–85). C1P regulates the invasion and migration of different types of cancer cells, including breast, lung, prostate, pancreas or leukemia cells (83). CerK phosphorylates cell-killing ceramides into C1P. The metabolic conversion of ceramide into SM is catalyzed by SMS. CerK and SMS can induce migration and invasion (61, 65). Some sphingolipid enzymes also have dual roles in different cancers as illustrated by CerK. Although CerK can regulate Rac1 activity and inhibit lamellipodium formation in A549 cells (60), it can promote migration in breast cancer (61).

## The role of sphingolipids as therapeutic targets in cancer

### Sphingolipid-based anti-tumor treatment strategy

Sphingolipids are currently recognized as multifaceted mediators in the biological and therapeutic fields of cancer. Therefore, they may become new targets for therapeutic application. Sphingolipid metabolism is becoming increasingly well studied in this field of cancer treatment. Several types of cytotoxic chemotherapeutic agents that cause ceramide accumulation, including doxorubicin, irinotecan, vincristine, and paclitaxel, have been clinically applied or undergoing clinical trials (43).

**Figure 2:**
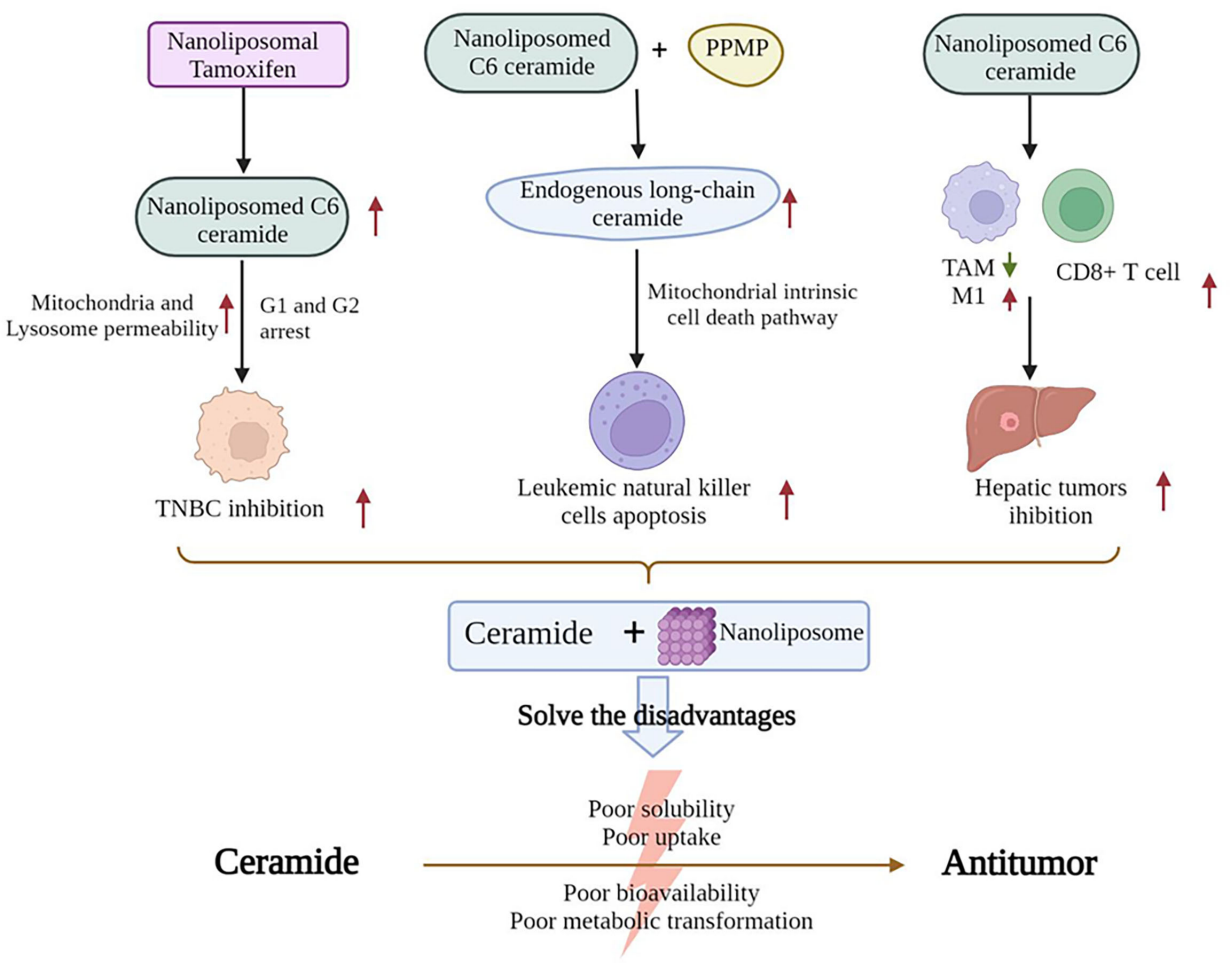
Nanoliposomes improve the antitumor activity of ceramides.

Although sphingolipids have been demonstrated to have an effective antitumor action, their poor solubility, uptake, bioavailability, and metabolic transformation have hindered the efforts to utilize their antitumor effect. Therefore, determining a way to improve efficacy is necessary for the development of ceramide-based therapy (**Figure 2**). For example, a previous work showed that in cultured TNBC cells, nanoliposomal tamoxifen enhances nanoliposomal C6-ceramide cytotoxicity. This reaction is accompanied by the induction of cell cycle arrest in G1 and G2 by caspase-induced DNA fragmentation. The permeability of the mitochondria and lysosomal membrane was enhanced at 18 and 2 h, respectively (86). The combination of C6-ceramide nanoliposomes with PPMP has been shown to lead to an increase in endogenous long-chain ceramide species, resulting in the apoptosis of leukemic natural killer (NK) cells *via* the mitochondrial endogenous cell death pathway (87). Moreover, in mice with hepatic tumors, the injection of nanoliposome-loaded LipC6 reduces the amount of tumor-associated macrophages and the ability of TAMs to inhibit the antitumor immune response. LipC6 also enhances the antitumor activity of tumor antigen-specific CD8^+^ T cells (88). A novel method for loading the short-chain C6 ceramide onto polyethylene glycol and polyethyleneimine co-conjugated ultra-small nano-GO can also result in high cancer-cell-killing potential in HCC (89).

### Anti-tumor treatment strategy based on sphingolipid enzymes

In addition to sphingolipids, sphingolipid enzymes have also become important targets for anti-tumor selection. SMase could be activated after stimulation by radiation and chemotherapeutic drugs, such as leukemia and glioma. The activated SMase catalyzes the hydrolysis of SM to produce ceramide. The produced ceramide would continue to activate different protein kinases and protein phosphatases (JNK and PKC), thus initiating the cascade signaling pathway, transmitting extracellular signals to cells, and then causing apoptosis. Therefore, the inhibition of enzymes that break down ceramide can alleviate chemotherapy resistance *via* sphingolipid regulation. The inhibitors of sphingolipid-metabolizing enzymes can be used as chemical sensitizers (37). Interestingly, SPHK1 has also been reported to promote the oxaliplatin resistance of HCC cells *via* the modulation of the Akt/GSK3β signaling pathway (16). So, the SPHK1 inhibitor maybe reverses oxaliplatin resistance.

Given that the dysregulation of sphingolipid enzymes can lead to the occurrence of cancer, the discovery of the optimal characterized inhibitors and activators targeting sphingolipid enzymes is important. At present, many inhibitors for different types of sphingolipid enzymes, such as Acid ceramidase (ASAH1), ACDase, CerK, SPHK, and GCS, have been discovered (90). B13 is a ceramide analogue that has been shown to be an effective *in vitro* ACDase inhibitor (30). B13 can accumulate cellular ceramides *in vivo*. It has been modified with a series of lysosomotrophic molecules, such as LCL521 (30), to address its difficult accumulation in lysosomes. NVP-231 is a CerK inhibitor. NVP-231 decreases cell viability, DNA synthesis and colony formation in a concentration-dependent manner. It can increase DNA fragmentation and induce apoptosis (91). Dimethylsphingosine is a methylated form of sphingosine and is the first SPHK inhibitor (90). The SphK inhibitor is called SK I-II. SPHK I-II simultaneously inhibits SPHK1 and SPHK2, as well as other targets, and SPHK inhibition with SPHK I-II decreases MDR breast cancer proliferation and viability (92). FTY720, a sphingosine analogue that induces S1P receptor down-regulation, is another SPHK1 inhibitor (fingolimod). It inhibits SPHK1 and blocks the activation of multiple targets of this enzyme. It directly or indirectly inhibits multiple intracellular targets responsible for cell proliferation, migration, and angiogenesis (93). Moreover, preclinical data support its possible use as an antineoplastic drug (94). The GCS inhibitors D-threo-1-phenyl-2palmitoylamino-3-morpholino-l propanol (PPMP) and D-threo-1-phenyl-2-decanoylamino-3-morpholino-propanol (PDMP) are ceramide analogues. A study on leukemic cell lines has shown that by blocking GCS with PDMP and PPMP simultaneously, cells become desensitized to daunomycin because of the antiapoptotic action of galactoside ceramide accumulation (95). Thus, the effect of sphingolipid enzymes and their inhibitors on cancer cells is an interesting topic that needs further studies.

### The role of sphingolipids in anti-cancer drug resistance

Drug resistance is a serious barrier to the successful therapy of patients with cancer. A large number of experiments have reported the mechanisms through which sphingolipids and their enzymes cause cancer drug resistance. Sphingolipid expression is also applied as a biomarker of drug resistance. The most widely known sphingolipid enzyme is GCS. This enzyme catalyzes the combination of glucose and ceramide to produce noncytotoxic GlcCer, which results in the decrement in cytotoxic Ceramide levels. The overexpression of GCS, which is particularly prominent in chemotherapy resistance, has been found in various cancers (96). In breast cancer, MDR1 overexpression is accompanied by other changes in genes, including GCS. MDR1 encodes P-glycoprotein, which could extrude anticancer drugs (97). Then, in breast cancer, high GCS expression is correlated with multidrug resistance (MDR), the poor prognosis of estrogen receptor (ER)+ tumors (30), and metastatic ER+ and human epidermal growth factor receptor 2 (HER2)+ tumors (Luminal B) (91). In ovcar-8 cancer cells, GCS transfection induces MDR1 overexpression and increases P-glycoprotein excretion in a dose-dependent manner. GSLs mediate MDR1 gene expression *via* cSrc and β-catenin signaling (97). In prostate cancer, SPHK1 inhibitors enhance the anticancer efficacy of enzalutamide (92). In addition, ceramide levels are lower in patients with chemoresistant leukemia than in patients with chemosensitive leukemia, whereas the activities of SMS and GCS in chemoresistant leukemia cells are higher than those in chemosensitive leukemia cells (98). Other studies have shown that sphingolipid enzymes, such as CDase and SPHK (99), are closely related to chemotherapy resistance.

## The relationship between sphingolipid metabolism and tumor immunotherapy

### Sphingolipids and tumor immunotherapy

A growing number of studies have demonstrated that metabolites in sphingolipid metabolism are closely associated with tumor immunity and that sphingolipids are involved in the interactions between cancer cells and the immune system. Sphingolipids are important components of the plasma membrane and can influence the function of receptors on the surfaces of immune cells. Metabolites are involved in the release of bioactive mediators, S1P, and ceramides, which influence the efflux and migration of lymphocytes into the tumor environment and regulate the critical pathways required for the activation of immune cells (100). The release of bioactive mediators, S1P, and ceramides can alter the function of immune cells, including Tregs lymphocytes, and macrophages, in the tumor microenvironment (TME), as well as alter the intracellular signaling pathways associated with immune cell activation or survival; such an alteration, in turn, may modulate the efficacy of antitumor immunotherapy (100). In a mouse model, ceramides inhibit the function of myeloid-derived suppressor cells (MDSCs) by activating lysosomal histone B and histone D; this effect leads to diminished autophagy and ER stress induction, thereby enhancing cytotoxic T lymphocyte function and inhibiting the growth of CMS4-met-derived soft tissue sarcoma tumors (77).

Other studies have reported that the metabolite C2-ceramide triggers apoptosis in melanoma cells by increasing PKCζ, as well as proinflammatory cytokines and signaling factors (101). C2-ceramide regulates immune cells in the TME and activates host protective immune responses through the up-regulation of PKCζ. In a PKCζ-dependent pathway, C2-ceramide repolarizes tumor-associated macrophages (TAMs) toward the M1 phenotype, thereby generating antitumor responses *in vitro* and *in vivo (* 101). Ceramide-mediated alterations in host-protective angiogenic factors and alterations in T helper 1 cell (Th1) and T helper 2 cell (Th2) cytokine levels lead to improvements in the TME. C2-ceramide tilts the immune component within the TME toward a proinflammatory state and differentially regulates immune cell–cancer cell interactions to limit tumor growth. At the same time, C2-ceramide can increase the proportion of activated CD8^+^ T-cells in the TME and up-regulate perforin and granzyme B, which kill tumors in response (101).

**Figure 3:**
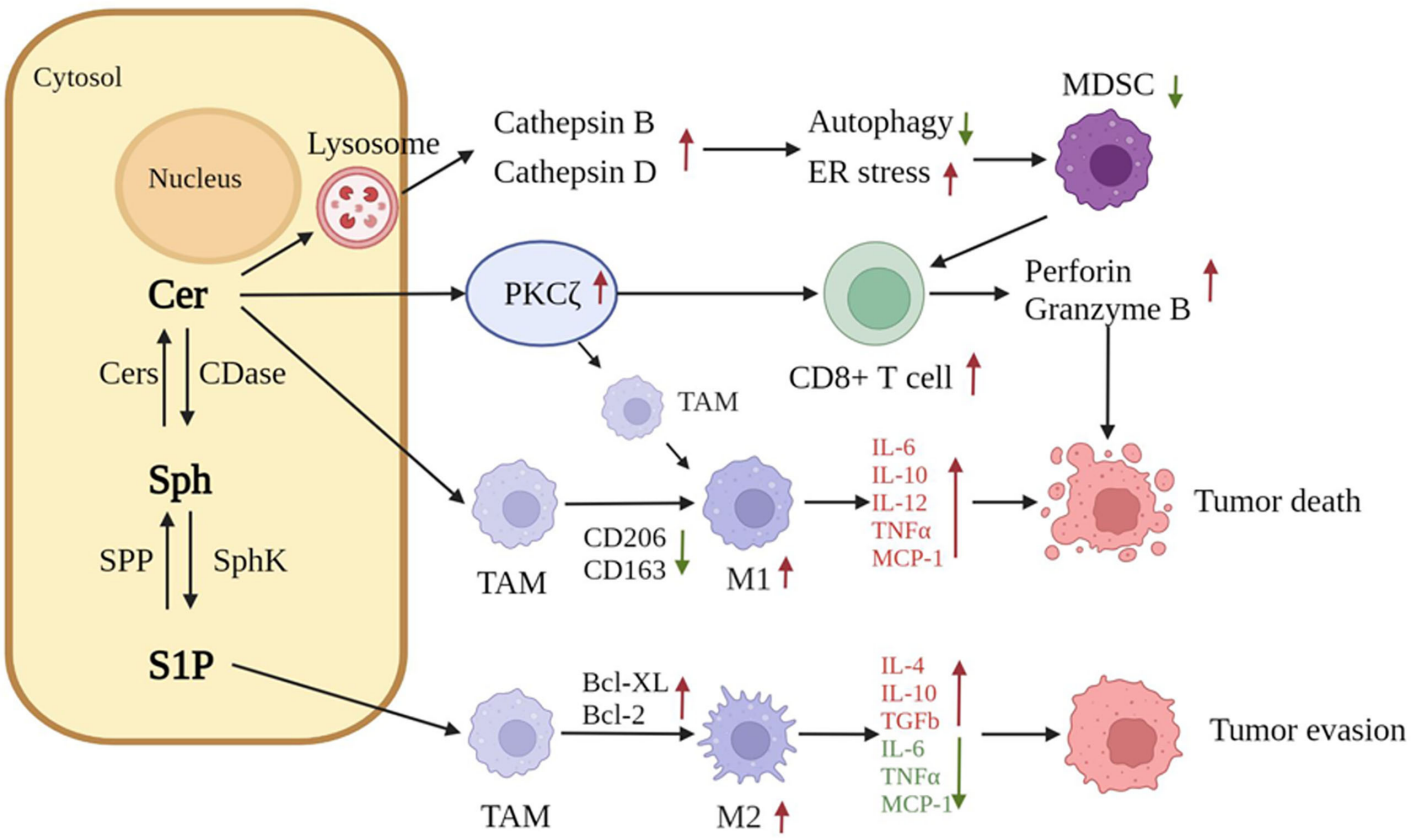
Influence of sphingolipids on the tumor microenvironment.

Ceramides modified with nanomaterials can further modulate the efficacy of tumor immunotherapy. Nanoliposomal C6-ceramide (LipC6) not only induces apoptosis and prevents HCC-induced immune tolerance in HCC cells, it also significantly decelerates the growth of liver cancer cells, enhances tumor-infiltrating CD8^+^ T cells, and inhibits tumor-resident CD4^+^ CD25^+^ forkhead box protein P3 (FoxP3^+^) Treg cells in combination with anti-CTLA4 Ab (102). Molecular studies have shown that combination therapy inhibits the transcription factors Krüppel-like factor 2, FoxP3 and CTLA4 (102). The dysregulation of sphingolipid metabolism is also frequently associated with cancer stem cells (103, 104) and consequently generates a series of tumor immune-related effects; this situation suggests that ceramide is a key player in cancer immunotherapy (104). Elevated levels of ceramides induce cancer cell death (105), enhance the host protective immune response, and induce cancer regression (101, 106). Elevated levels of S1P induce an increase in the antiapoptotic proteins Bcl-2 and Bcl-XL in macrophages and the M2-type polarization of macrophages, which can also recruit immune cells to immune tissues for immune action (107, 108). S1P released from apoptotic or tumor cells, in combination with its specific receptor (S1PR1), induces the recruitment of circulating monocytes to the TME (109). α-Galactose ceramide (α-GalCer) has been noted for its role in activating invariant natural killer T (iNKT) lymphocytes and has been dicscovered to have a beneficial effect on the immune system. It has been noticed for its role in activating iNKT lymphocytes and has been found to exert oncogenic effects in a variety of tumors by inducing effective iNKT cells. The addition of PD-1 blockers to α-GalCer treatment prevents iNKT cell loss, resulting in the skewing of CD4^+^ cells toward Th1. This situation suggests that the combination of α-GalCer and immune checkpoint blockers may be a promising approach for improving the efficacy of tumor immunotherapy (110). In conclusion, sphingolipids are closely related to tumor immunity, and altered levels of sphingolipid metabolism are important factors influencing tumor immunotherapy (**Figure 3**).

### Correlation between enzymes in the sphingolipid pathway and tumor immunotherapy

Now that we understand that sphingolipids are closely associated with tumor immunotherapy, we are eager to learn the exact mechanisms underlying the important roles of SMases in association with tumor immunotherapy. In different T cell subpopulations in mice and humans, sphingolipids are metabolized by varying degrees into ceramides that, in turn, participate in immune regulation and influence T cell activation, differentiation, and effector functions (111). ASMase (mouse: Asm; human: ASM) is a key mediator of sphingolipid catabolism. SMase deficiency impairs T-cell function, whereas increasing SMase activity increases T-cell function, thereby improving the efficacy of anticancer immunotherapy. These phenomena suggest that increasing ASM activity in T cells through drugs may enhance T cell-mediated immunity and may thus improve tumor killing (111).

**Figure 4:**
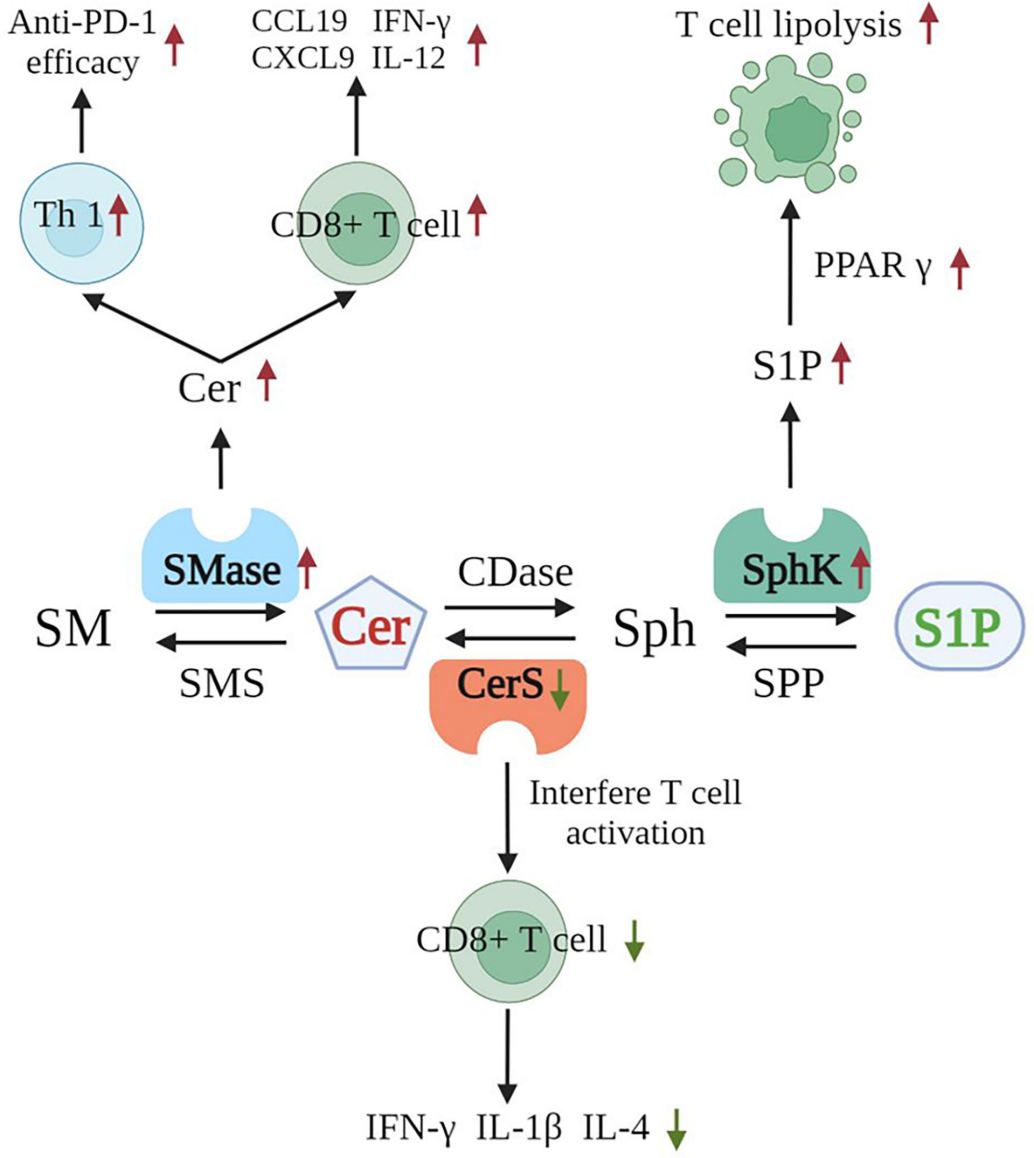
Functions of sphingolipid enzymes in the immune system.

Ceramide synthase 5 is one of six enzymes that catalyze the production of ceramides from sphingosine. Ceramides are an essential component of cell membranes and act as signaling molecules. CerS5 knockout mice have significantly reduced CD8^+^ T cells in the colonic epithelium. This decrement is accompanied by the reduced expression of IL-1β, IFNγ, and IL-4. The mice show increased susceptibility to colon cancer. *In vitro* studies have found that the knockdown of ceramide synthase 5 in T cells impairs T cell activation (112). Moreover, SPHK1 produced S1P is directly linked to the activity of the lipid transcription factor peroxisome proliferator-activated receptor γ, which subsequently regulates lipolysis in T cells. The inhibition of SPHK1 increases the antitumor activity of T cells against murine melanoma (113). Alkaline ceramidase 3 (Acer3) mediates the immune response by regulating C18:1-ceramide levels in the cells of the innate immune system, and Acer3 deficiency increases the levels of proinflammatory cytokines in colonic epithelial cells; this effect may subsequently regulate the development and progression of intestinal cancer (114). Then, neutral SMase 2 (nSMase2) is encoded by SMPD3 and catalyzes the breakdown of SM to produce ceramide. In mouse models of melanoma and breast cancer, the overexpression of wild-type nSMase2 increases anti-PD-1 efficacy. This increase is associated with an enhanced Th1 response. In wild-type mice, nSMase2-overexpressing tumors accumulate ceramide and CD8^+^ tumor-infiltrating lymphocytes, which are associated with increased levels of transcripts encoding IFNγ and CXCL9. *In vitro*, small extracellular vesicles from melanoma cells overexpressing wild-type nSMase2 enhance the expression of IL12, CXCL9, and CCL19 in bone marrow-derived dendritic cells, suggesting that melanoma nSMase2 triggers Th1 polarization at the earliest stages of the immune response (115). This situation suggests that the modulation of sphingolipid metabolism-related enzymes may alter the efficacy of tumor immunotherapy effectively (**Figure 4**). Hope for the discovery of additional effective targets for modulating tumor immunotherapy has been found with the progression of the research on sphingolipid metabolism-related enzymes in relation to tumor immunity.

## Natural products for sphingolipid-based anticancer therapeutics

Natural products medicine has been practiced for thousands of years all over the world and has played an critical role in the treatment and prevention of different diseases throughout history (116). Given their safety and low side effects, natural products are used as alternative cancer therapies. Numerous natural products have various biological activities and low toxicity at the same time and are currently widely accepted as an excellent source of anticancer drugs (117). Previous studies have shown that some herbal compounds can treat and prevent cancer from recurring and metastasizing and can thus prolong the survival of postoperative patients with cancer. When applied in the early stage of cancer, active ingredients can cure pre-cancerous conditions and decrease the rate of cancer occurrence (118). With the transformation of modern medical concepts from treatment to strengthening the physique, the advantages of natural products have become more apparent than before. Thus, as indicated in **Table 3**, natural products can be used as platforms for the design of sphingolipid-related anticancer drugs.

**Table 3:** Summary of anticancer therapies of Natural compounds.

Natural compounds	Targets inhibited	Comments	Mechanism of action	Refs
Sanguinarine	ACDase and GCS	natural product from *Sanguinaria canadensis*, *Chelidonium majus* and *Macleaya cordata*	SNG inhibited ACDase and GCS enzymes, subsequently leading to Cer generation and apoptosis	(119)
Jaspine B	SMS	natural product from marine sponge *Jaspis sp*	It was able to dose- and time-dependently decrease the viability of murine B16 and human SK-Mel28 melanoma cells via triggering of apoptosis	(120)
Myriocin	SPT	natural product from *Myriococcum ablomyces*	•It induced growth inhibition in melanoma cells by inducing cell cycle arrest in the G2/M phase and increased the expression of antiproliferative genes p53 and p21 •It induced death of lung cancer cells via apoptosis and synergistically inhibited cancer cell growth when given in combination with anti-tumor drugs docetaxel and cisplatin	(73, 121, 122)
S-15183a/b	SK1	natural product from *Zopfifiella inermis*	S-15183a is cell permeable with selectivity for the PKC, PI3K. S-15183b inhibit SK1 in a dose-dependent manner with an IC50 of 1.6µM	(123)
F-12509A	SK1/SK2	natural product from *Trichopeziella barbata*	Inhibition of SK1 and SK2 by F-12509A overcame chemoresistance in chemosensitive and chemoresistant HL60 cells. It is selective to PI3K and PKC, but it weakly inhibit CerK	(123, 124)
B-5354c	SK1/SK2	natural product from marine bacterium	The sensitivity of LNCaP and PC-3 cells to docetaxel was enhanced. Combined action with irinotecan on the mouse model of prostate tumor in-situ can reduce the tumor size	(125–127)
Englerin A	SMase	natural product from the Tanzanian plant *Phyllanthus engleri*	Englerin A inhibited renal carcinoma cells by significantly altering lipid metabolism and increased ceramide levels	(128)
Tricin	SPHK	natural product from *rutabaga*	Tricin inhibited the tumor growth mainly by suppressing PRKCα/SPHK/S1P signaling and antiapoptotic signaling.	(129)
Resveratrol	DES	natural product from *Polygonum cuspidatum Sieb.et Zucc.*	Resveratrol induces autophagy in gastric cancer cells (HGC-27) by inhibiting dihydroceramide desaturase and increasing dihydroceramide.	(130)

ACDase, acid ceramidase; GCS, glucosylceramide synthase; SNG, sanguinarine; Cer, ceramide; SMS, sphingomyelin synthetase; SPT, serine-palmitoyltransferase; SK1, sphingosine kinase 1; SK2, sphingosine kinase 2; PKC, protein kinase C; PI3K, the phosphatidylinositol3-kinase; CerK, ceramide kinase; SMase, sphingomyelinase; ASM, acid sphingomyelinase; QYSLD, Qi-Yu-San-Long Decoction; SPHK, sphingosine kinase; PRKCα, Protein Kinase C Alpha; S1P, Sphingosine-1-phosphate; DES, dihydroceramide desaturase.

Many natural compounds isolated from bacteria and fungi possess SPHK inhibition activity. B-5354c, which is produced by a marine bacterium, inhibits SPHK1 and SPHK2 with similar efficiencies (131) and acts on sphingosine in a noncompetitive manner. B-5354c can reduce the size of PC-3 tumors *in vivo* when combined with the chemotherapy drug irinotecan and can sensitize PC-3 and LNCaP prostate cancer cells to chemotherapy drugs, such as docetaxel and camptothecin (132). B-5354c has been further demonstrated to act as a chemosensitizer of PC-3 cells when used in conjunction with docetaxel (133). F-12509A (125, 126), extracted from *Trichopeziella barbata*, and S-15183a/b (126), extracted from *Zopfifiella inermis*, are also natural products that have been found to inhibit SPHK. Jaspine B is an anhydrophytosphingosine, extracted from marine sponge *Jaspis sp*, with potential antineoplastic property. It can increase the concentration of ceramides within cells by inhibiting SMS. Jaspine B can also induce apoptosis in human SK-Mel28 melanoma and murine B16 cells (127). Myriocin, extracted from *Myriococcum ablomyces*, specifically inhibits SPT, leading to the suppression of cell growth by triggering the cell cycle in melanoma cells (124) and significantly inhibiting tumor formation in melanoma mice (123). Myriocin combined with docetaxel and cisplatin can also inhibit the growth of cancer cells (120). In prostate cancer, sanguinarine, extracted from *Sanguinaria canadensis*, *Chelidonium majus* and *Macleaya cordata*, inhibits ACDase and GCS enzymes, subsequently resulting in ceramide generation and apoptosis (121).Tricin, a component extracted from rutabaga, exerts proliferation-inhibiting, proapoptotic, and migration-inhibiting effects on Lewis lung cancer cells. It inhibits tumor growth mainly by inhibiting PRKCA/SPHK/S1P signaling and antiapoptotic signaling (73). Resveratrol, extracted from *Polygonum cuspidatum Sieb.et Zucc*., induces autophagy in gastric cancer cells (HGC-27) by inhibiting dihydroceramide desaturase and increasing dihydroceramide (122). Moreover, resveratrol also triggers anti-proliferative and apoptotic effects in FLT3-ITD-positive acute myeloid leukemia cells through inhibition of ceramide catabolic enzymes (119, 134). Vincristine is a vinca alkaloid that can be obtained from the Madagascar periwinkle *Catharanthus roseus (* 135, 136). The combination of vincristine and SPHK2 inhibitor ABC294640 significantly enhanced the inhibition of SPHK2 and further inhibited the survival and proliferation of T-cell acute lymphoblastic leukemia (T-ALL) cells (129).

Overall, targeting sphingolipid enzymes and altering sphingolipid metabolism can lead to promising anticancer effects. In addition, natural products are a primary source of potential anticancer agents and can thus improve and increase the effectiveness of cancer therapies. The discovery and clarification of their mechanisms of action are urgently needed.

## Summary and future perspectives

Sphingolipids have a broad scope of biological functions. Numerous discoveries have elucidated the different roles and mechanisms of sphingolipids in cancers. The unique anticancer or oncogenic functions of sphingolipids in cancers rely on microenvironmental conditions, cell types, and immune status. The rapid metabolism and signaling of sphingolipids within biological membranes have a strong influence on the regulation of cell death and survival. Further studies are needed to determine how sphingolipid enzymes regulate proliferation in cancer systems. Therefore, additional frontier molecular and analytical tools and technologies should be developed and applied in sphingolipid-related anticancer investigations. Although a large number of preclinical trials have shown good results, FDA-approved drugs targeting sphingolipids in cancer treatment remain lacking. Natural drugs, such as TCMs, have made many contributions to the study of sphingolipid metabolism. In the future, strengthening the cooperation in the research and clinical practice of sphingolipids through the integration of Chinese and Western medicine will be necessary and is expected to increase the effectiveness of tumor diagnosis and treatment.

Endogenous ceramides are synthesized through the *de novo* pathway, which begins with palmitoyl-CoA and the condensation of the amino acid serine to form 3-ketosphinganine *via* SPT, leading to the synthesis of dihydroceramide by CerS. Dihydroceramides are then converted into ceramides by DES. Ceramides may also be produced through the breakdown of SMs or the degradation of complex sphingolipids under the action of SMase and GCase. In addition, the salvage pathway can generate endogenous ceramides. Sph, a product of sphingolipid breakdown metabolism, can be reanylated by CerS, resulting in the production of ceramides. Once produced, ceramides can briefly accumulate or be converted into different sphingolipids. Ceramides are phosphorylated by CerK to produce C1P and utilized as a substrate by CDase to liberate Sph, which is phosphorylated to generate S1P. Ceramides can also be reversibly metabolized into SM, that is, phosphorylcholine and ceramide generate SM under the action of SMS. GCS can remove ceramides by catalyzing the conversion of ceramides into glycosheath ester. Ceramides can also be converted into GalCer by CGT. (SPT, serine palmitoyl transferase; CerS, ceramide synthases; DES, dihydroceramide desaturase; SMs, sphingomyelins; SMase sphingomyelinases; GCase, glucosylceramidases; Sph, Sphingosine; CerK, ceramide kinase; C1P, ceramide CDase, 1-phosphate; ceramidase; S1P, sphingosine-1-phosphate; SM, sphingomyelin; SMS, phingomyelin synthetase; GCS, Glucoceramide synthetase; GalCer, galactosylceramide; CGT, galactosylceramide transferase.)

Ceramides have antitumor effects but have poor solubility, absorption, bioavailability, and metabolic conversion. Nanoliposomes can help address these disadvantages of ceramides. First, nanoliposomal tamoxifen can enhance the cytotoxicity of nanoliposomal C6-ceramide in TNBC cells, induce cell cycle arrest in the G1 and G2 phases, and increase the permeability of mitochondrial and lysosomal membranes, thus killing TNBC cells. Nanoliposomal C6-ceramide, when combined with PPMP, increases the amount of endogenous long-chain ceramides and increases the apoptosis of leukemic NK cells *via* the endogenous mitochondrial cell death pathway. Nanoliposomal C6-ceramide reduces the number of TAMs, induces the differentiation of TAMs into the M1 phenotype, increases CD8^+^ T cell activity, and suppresses the proliferation of hepatocellular tumors. (TNBC, Triple-Negative Breast Cancer; PPMP, [2]1-phenyl-2-palmitoylamino-3-morpholino-1-propanol; TAM, tumor-associated macrophage).

Ceramides activate lysosomal histone B and histone D, leading to the reduction in autophagy and the induction of endoplasmic reticulum stress. These effects further inhibit the function of MDSCs and enhance the activity of cytotoxic T lymphocytes. C2-ceramide up-regulates PKCζ, repolarizes TAM toward the M1 phenotype, increases the activation ratio of CD8^+^ T cells, and up-regulates the secretion of perforin and granzyme B to kill tumors. S1P induces the conversion of TAM into M2 and promotes the secretion of IL-4, IL-10, and TGFb, thus leading to tumor evasion. (Cer, ceramide; S1P, sphingosine 1 phosphate; MDSCs, myeloid-derived suppressor cells; TAM, tumor-associated macrophage).

Several enzymes are closely related to the effects of tumor immunotherapies. SMase catalyzes the production of ceramide from SM, triggering Th1 polarization, and enhancing anti-PD-1 efficacy. SMase induces ceramide accumulation, thus enhancing the activity of CD8^+^ tumor-infiltrating lymphocytes and increasing the expression levels of IFNγ, IL12, CXCL9, and CCL19. S1P is catalyzed by SphK directly in relation to the activity of the lipid transcription factor peroxisome proliferator-activated receptor γ, which promotes the lipolysis of T cells. Cer (red) indicates promoted immune killing of tumors, and S1P (green) indicates immunity inhibition, allowing tumors to escape. (Cer, ceramide; S1P, sphingosine 1 phosphate; SMase, sphingomyelinase; Th1, T helper 1; Cers, ceramide synthase; SphK, sphingosine kinase; PPAR γ, peroxisome proliferator-activated receptor γ).

## Author contributions

(I) Conception and design: EL-HL, R-ZL, P-YY. (II) Administrative support: LL, EL-HL; (III) Manuscript writing: All authors; (IV) Final approval of manuscript: All authors.

## Funding

This work is supported by the 2020 Guangdong Provincial Science and Technology Innovation Strategy Special Fund (Guangdong-Hong Kong-Macau Joint Lab) (No: 2020B1212030006). This work is also supported by Macao Science and Technology Development Fund (Project no: 0096/2018/A3, 0056/2020/AMJ), and from Dr. Neher's Biophysics Laboratory for Innovative Drug Discovery (001/2020/ALC). This work is also supported by 2020 Young Qihuang scholar granted to Prof. Elaine Leung from the National Administration of Traditional Chinese Medicine.

## Conflict of interest

The authors declare that the research was conducted in the absence of any commercial or financial relationships that could be construed as a potential conflict of interest.

## Publisher’s note

All claims expressed in this article are solely those of the authors and do not necessarily represent those of their affiliated organizations, or those of the publisher, the editors and the reviewers. Any product that may be evaluated in this article, or claim that may be made by its manufacturer, is not guaranteed or endorsed by the publisher.

## Abbreviations

α-GalCer, α-Galactose ceramide; ACDase, acid ceramidase; ASMase, acid sphingomyelinase; Acer3, Alkaline ceramidase 3; alk-SMase, alkaline sphingomyelinase; CDase, ceramidase; Cer, ceramide; C1P,ceramide 1-phosphate; UGT8, ceramide galactosyltransferase; CerK, ceramide kinase; CerS, ceramide synthase; DES, dihydroceramide desaturase; LASS, dihydroceramidsynthases; PDMP, D-threo-1-phenyl-2-decanoylamino-3-morpholino-propanol; PPMP, D-threo-1-phenyl-2palmitoylamino-3-morpholino-l propanol; FoxP3+, forkhead box protein P3; GlcCer, glucosylceramide; GSLs, glycosphingolipids; GCS, glucosylceramide synthase; LipC6, Nanoliposomal C6-ceramide; NSMase, neutral sphingomyelinase; NSMase2, neutral sphingomyelinase 2; PPAR γ, peroxisome proliferator-activated receptor γ; SPT, serine palmitoyl transferase; SMase, sphingomyelinases; SM, sphingomyelin; SMS, sphingomyelin synthetase; S1P, sphingosine 1-phosphate; SPL, sphingosine 1-phosphate lyase; SphK/SPHK, sphingosine kinase; SPHK1, sphingosine kinase 1; CRC, colorectal cancer; HCC, hepatocellular carcinoma; HNSCC, human head and neck squamous cell carcinoma; iNKT, invariant natural killer T; MDSCs, myeloid-derived suppressor cells; NK, natural killer; Th1, T helper 1 cell; Th2, T helper 2 cell; TNBC, triple negative breast cancer; TAM, tumor-associated macrophage. ER, estrogen receptor; HER2, human epidermal growth factor receptor 2; LN, lymph node; MDR, multidrug resistance; TME, tumor microenvironment; OS, overall survival.
